# Translocated duplication of a targeted chromosomal segment enhances gene expression at the duplicated site and results in phenotypic changes in *Aspergillus oryzae*

**DOI:** 10.1186/s40694-018-0061-6

**Published:** 2018-10-03

**Authors:** Tadashi Takahashi, Masahiro Ogawa, Atsushi Sato, Yasuji Koyama

**Affiliations:** Noda Institute for Scientific Research, 399 Noda, Noda City, Chiba Pref 278-0037 Japan

**Keywords:** Targeted chromosomal duplication, Translocated duplication, Break induced replication, *Aspergillus oryzae*, Phenotypic change, *prtT*, Screening, Molecular breeding, Biotechnology, Genome engineering, Gross genome editing, Protease

## Abstract

**Background:**

Translocated chromosomal duplications occur spontaneously in many organisms; segmental duplications of large chromosomal regions are expected to result in phenotypic changes because of gene dosage effects. Therefore, experimentally generated segmental duplications in targeted chromosomal regions can be used to study phenotypic changes and determine the functions of unknown genes in these regions. Previously, we performed tandem duplication of a targeted chromosomal segment in *Aspergillus oryzae*. However, in tandem chromosomal duplication, duplication of chromosomal ends and multiple chromosomal duplication are difficult. In this study, we aimed to generate fungal strains with a translocated duplication or triplication of a targeted chromosomal region via break-induced replication.

**Results:**

Double-strand breaks were introduced into chromosomes of parental strains by treating protoplast cells with I-SceI meganuclease. Subsequently, strains were generated by nonreciprocal translocation of a 1.4-Mb duplicated region of chromosome 2 to the end of chromosome 4. Another strain, containing a triplicated region of chromosome 2, was generated by translocating a 1.4-Mb region of chromosome 2 onto the ends of chromosomes 4 and 7. Phenotypic analyses of the strains containing segmental duplication or triplication of chromosome 2 showed remarkable increases in protease and amylase activities in solid-state cultures. Protease activity was further increased in strains containing the duplication and triplication after overexpression of the transcriptional activator of proteases *prtT*. This indicates that the gene-dosage effect and resulting phenotypes of the duplicated chromosomal region were enhanced by multiple duplications, and by the combination of the structural gene and its regulatory genes. Gene expression analysis, conducted using oligonucleotide microarrays, showed increased transcription of a large population of genes located in duplicated or triplicated chromosomal regions.

**Conclusion:**

In this study, we performed translocated chromosomal duplications and triplications of a 1.4-Mb targeted region of chromosome 2. Strains containing a duplication of chromosome 2 showed significant increases in protease and amylase activities; these enzymatic activities were further increased in the strain containing a triplication of chromosome 2. This indicates that segmental duplications of chromosomes enhance gene-dosage effects, and that the resulting phenotypes play important phenotypic roles in *A. oryzae*.

**Electronic supplementary material:**

The online version of this article (10.1186/s40694-018-0061-6) contains supplementary material, which is available to authorized users.

## Background

In eukaryotic organisms, duplications, translocations, reversions, and deletions of chromosomal regions occur spontaneously. For example, in *Saccharomyces cerevisiae*, duplication and translocation of chromosomes frequently occurs via Ty transposable element sequences, which are repeat sequences widely distributed in yeast chromosomes [1]. Similarly, previous studies of mammalian cells indicated that duplications, translocations, and deletions of chromosomes occur via repeat sequences such as Alu and L1 [2]. Changes in the chromosomal state often induce significant alterations in gene expression and result in phenotypic changes. In humans and other mammals, chromosome duplication or translocation events are reported to be among the causes of genetic diseases and cancers [3, 4].

**Fig. 1:**
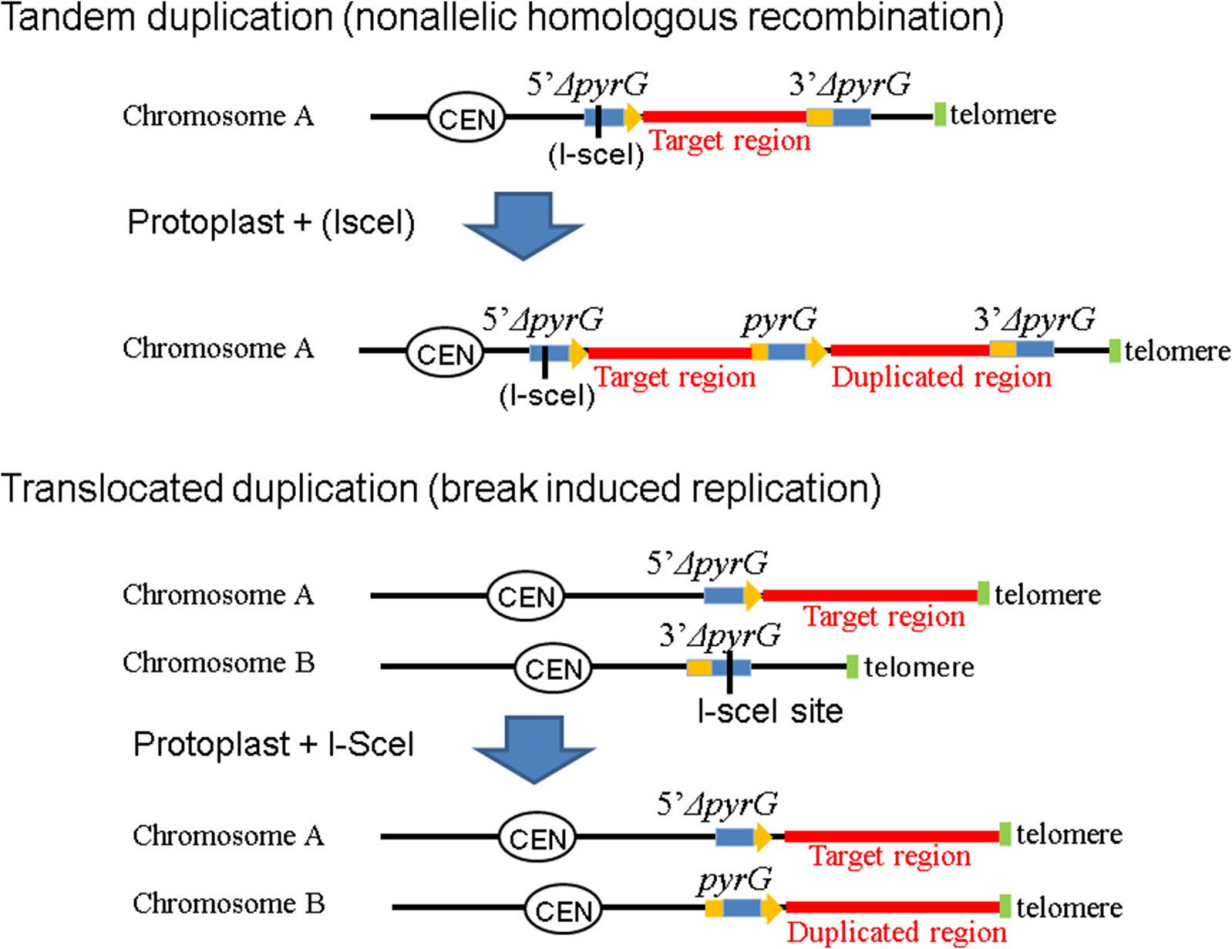
Types of chromosomal duplication. The top part of the figure shows a schematic representation of tandem chromosomal replication by nonallelic homologous recombination, where the duplicated region was arrayed in the same chromosome. The bottom of the figure shows a schematic representation of translocated duplication by break-induced replication, where the duplicated region was translocated to the other chromosome

The koji molds *Aspergillus oryzae* and *Aspergillus sojae* are filamentous fungi that are used in the fermentation of soy sauce and sake, and in production of industrial enzymes. The complete genome sequences of *A. oryzae* and *A. sojae* have been determined [5, 6]. Previously, genetic tools such as gene targeting [7, 8] and large chromosomal deletions [9, 10], have been developed to analyze the nature of *A. oryzae* and *A. sojae.* However, in koji molds, the functions of many genes remain unknown. Because of the presence of endogenous multiple orthologous genes, disrupting individual genes in strains of koji molds does not always generate clear phenotypic changes [5]. Overexpression of individual genes via a strong promoter can also cause reduced growth phenotypes [11]. However, strains with duplicated chromosomes can show phenotypic changes via increased dosage of unidentified genes [12]. By examining the genome of targeted chromosomal segments and phenotypic changes in the strains, we can determine the role of unknown genes corresponding to these phenotypic changes. In this study, we generated targeted segmental chromosomal duplications in *A. oryzae* and examined the phenotypic effects of these duplications. There are two common types of segmental chromosomal duplications: tandem duplication and translocated duplication (Fig. 1). In a tandem duplication, duplicated regions are arranged next to each other on the same chromosome by nonallelic homologous recombination (Fig. 1, top). In translocated duplications, chromosomal duplications are translocated onto another chromosome by break-induced replication (Fig. 1, bottom). We previously generated *A. oryzae* strains containing targeted tandem chromosomal duplications using protoplasts of the parental strain, in which artificially generated consensus sequences (partly deleted selection marker) were introduced into both ends of the duplication target region [12]. If the copy number of the duplication target region is increased, such as in triplication, the phenotypic change in the strain may be further enhanced. However, to maintain stability of a tandem chromosomal duplication, a selection marker must be located at the junction between the two duplicated segments. In *A. oryzae*, this prevents removal of the duplicated region by recombination events occurring between the duplicated homologous sequences [12]. Therefore, it is technically difficult to increase the copy numbers of genes located in duplicated regions in tandem chromosomal duplications. Moreover, it is difficult to duplicate chromosomal ends in tandem duplications because a selection marker positioned at the chromosomal end is unstable and lost during regeneration (Takahashi et al. unpublished data). In contrast, generating multiple translocated chromosomal duplications is theoretically possible because the potential for recombination between the two duplicated regions is low in translocated chromosomal duplications, and selective pressures are not required to maintain the translocation. Furthermore, duplication of chromosomal ends is straightforward in translocated duplication because a selection marker is not required at chromosomal ends. However, no previous studies have generated translocated duplications of targeted chromosomal regions in filamentous fungi such as *A. oryzae*. Therefore, we generated a translocated duplication of a targeted chromosomal segment in *A. oryzae*.

The break-induced replication (BIR) mechanism causes translocated chromosomal duplications in yeast [13–15]. However, no previous studies have shown that the same mechanism can be used in filamentous fungi such as *A. oryzae*. To cause BIR in *A. oryzae*, it is necessary to artificially introduce double-strand breaks (DSBs) into the chromosomes (Fig. 1, bottom). In most studies on yeast, DSBs are introduced after the expression of genes encoding endogenous yeast homothallic switching endonuclease (*HO*) or I-SceI endonuclease (*SCEI*). In contrast, chromosome modifications have been performed in *A. oryzae* using polyethylene glycol (PEG)-mediated introduction of enzymes into protoplast cells [12, 16–18]. Therefore, we generated chromosomal duplications by directly treating protoplast cells with I-SceI meganuclease and monitoring the resulting chromosomal DSBs. This approach eliminated the need for I-SceI expression and produced a translocated chromosomal duplication strain containing 1.4-Mb segment of the targeted chromosomal region. To the best of our knowledge, this is the first study to generate a targeted translocated chromosomal duplication using BIR and analyze the effect of chromosomal duplication on gene expression in filamentous fungi. Phenotypes of the strain resulting from this chromosomal duplication show increased activities of protease and amylase, indicating that this method can be used in functional analysis and molecular breeding of *Aspergillus* strains.

## Results

### Translocated duplication of targeted segment of chromosome 2 onto chromosome 4 and a strain bearing translocated triplication

**Fig. 2:**
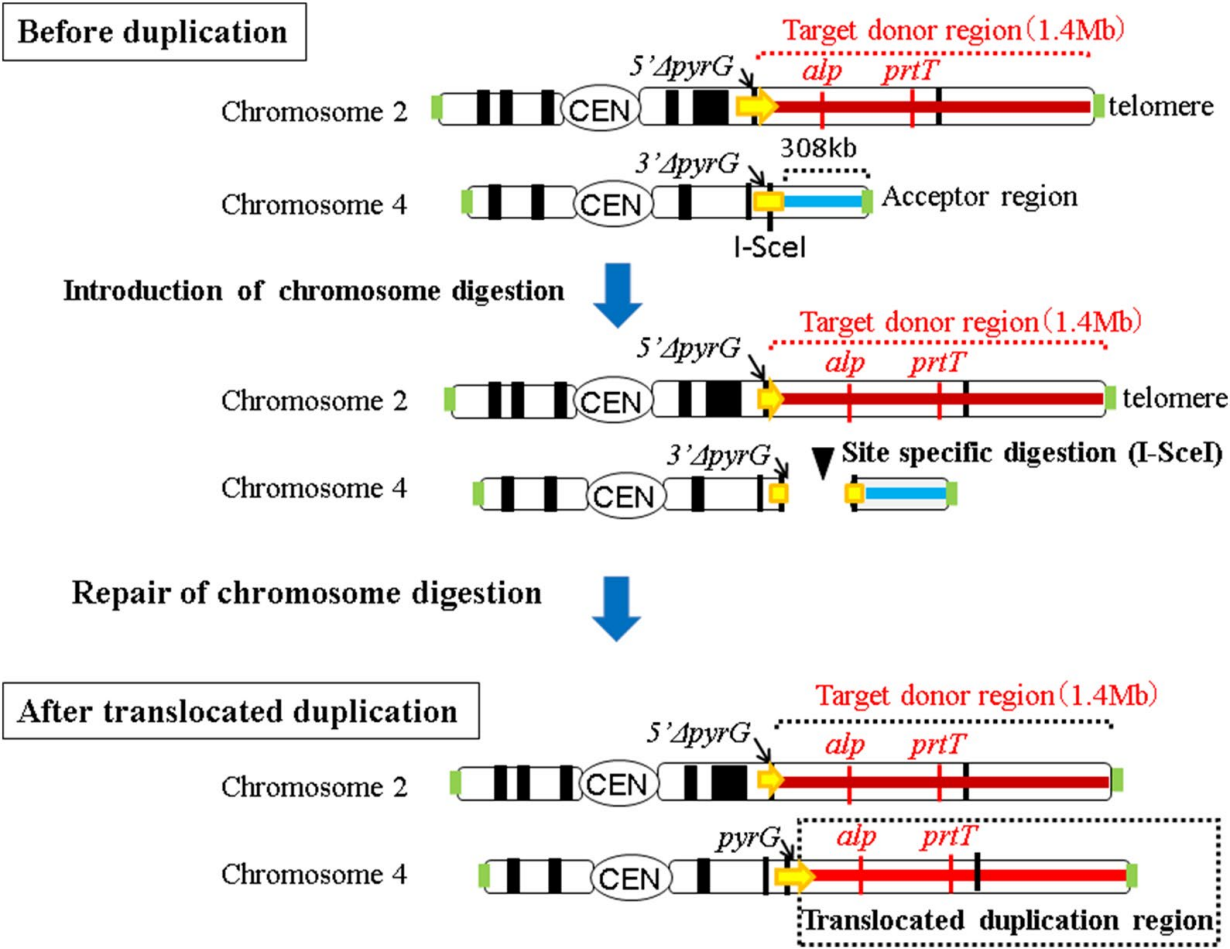
A schematic showing translocated duplication of chromosome 2 in a strain of *A. oryzae*. A 1.4-Mb target donor region of chromosome 2 was duplicated and translocated to the acceptor region of chromosome 4 using break-induced replication

**Fig. 3:**
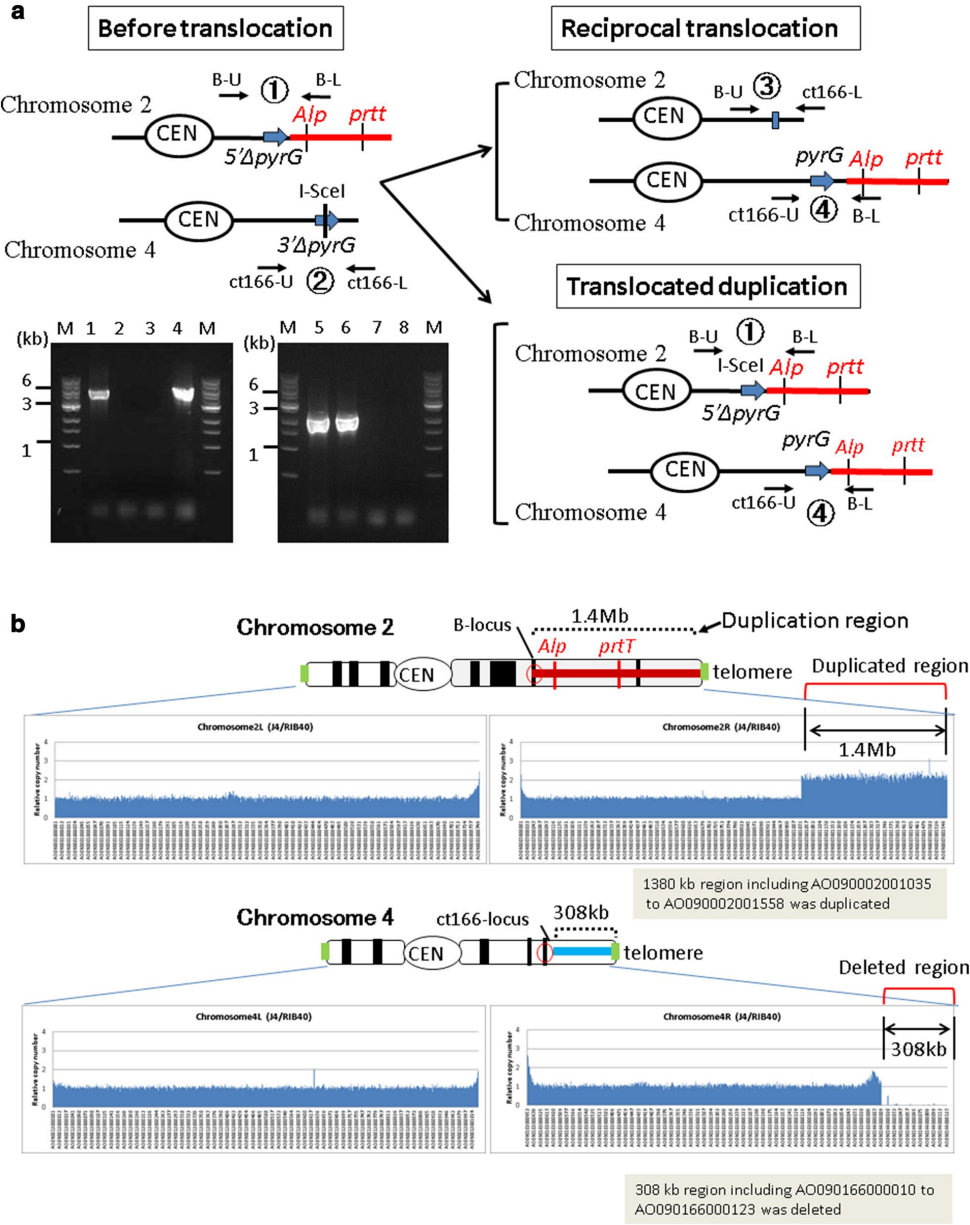
**a** Confirmation of translocated duplication by PCR. Targeted translocated duplication of a 1.4-Mb region of chromosome 2 to chromosome 4 was confirmed by PCR. *Lane 1*. B–U, B–L; *Lane 2*. ct166-U, ct166-L; *Lane 3*. B–U, ct166-L; *Lane 4*. ct166-U, B–L (*Lanes 1–4*. translocated duplication strain J4); *Lane 5*. B–U, B–L; *Lane 6*. ct166-U, ct166-L; *Lane 7*. B–U, ct166-L; *Lane 8*. ct166-U, B–L (*Lanes 5–8*. Wild-type strain). **b** Confirmation of translocated duplication by comparative genome hybridization (CGH) arrays. Vertical bars show ratios of signal intensities for probes of the strain bearing translocated duplication (J4) relative to those of the control strain (RIB40). Ratios of hybridization signals in the 1.4-Mb region of chromosome 2 were twice those of the other region, indicating that the 1380-kb region of chromosome 2, including AO090002001035 to AO090002001558, was duplicated. The ratio of hybridization signals in the 308-kb region of chromosome 4 was nearly zero, indicating that the 308-kb region of chromosome 4, including AO090166000010 to AO090166000123, was deleted

We previously constructed strains containing targeted tandem chromosomal duplications [12]. Chromosome 2 of *A. oryzae* includes genes encoding alkaline protease and alpha-amylase, and their respective regulatory genes *prtT* and *amyR*, which are important for fermentation. Accordingly, strains containing a 700-kb tandem chromosomal duplication in chromosome 2 showed increased protease and amylase activities under solid-state culture conditions [12]. In the present study, we duplicated a 1.4-Mb region of chromosome 2 and translocated this region onto the end of chromosome 4 (Fig. 2). Successfully translocated duplications can increase the activities of protease and amylase in solid-state cultures, as shown previously by tandem duplication of chromosome 2 [12]. In subsequent analyses using BLASTN, no I-SceI recognition sequence was found in the chromosome of *A. oryzae* strain RIB40 [17]. To translocate the 1.4-Mb region of chromosome 2 onto the end of chromosome 4, we placed the *5′ΔpyrG* marker (targeted integration of the basic unit of 5′*ΔpyrG* and subsequent 5-fluoroorotic acid [5-FOA] selection) adjacent to the target donor chromosomal region. The *3′ΔpyrG* marker (targeted integration of the basic unit of 3′*ΔpyrG* and subsequent 5-FOA selection), which included the I-SceI recognition sequence, was placed adjacent to the acceptor chromosomal region (Fig. 2, top). The resulting parental strain was then used to generate translocated duplication of the targeted region of chromosome 2. For these procedures, protoplast cells of the parental strain were prepared and gently treated with I-SceI meganuclease and PEG. The treated protoplasts were then incubated at 30 °C on Czapek-Dox minimum medium (CZ) plates containing 1.2 M sorbitol. After more than 2 weeks of incubation, we observed a colony that was regenerated from treated protoplasts. The frequency of the number of regenerated colonies was approximately 10^−8^ per cell. To confirm that translocated duplication was achieved in the regenerated strain, genomic DNA of the regenerated strain was extracted and analyzed by PCR using primers targeting the border of the translocated region (Fig. 3a).

We detected amplification of the 5-kb DNA fragment using combinations of primers targeting the translocated position (primers ct166-U and B-L in lane 4 of Fig. 3a) and original position (primers B-U and B-L in lane 1 of Fig. 3a) in the regenerated strain. In contrast, amplification of the 3-kb fragment was detected only by using the primer combination corresponding to the original position (primes B-U and B-L in lane 5 of Fig. 3a, and primers ct166-U and ct166-L in lane 6 of Fig. 3a) in the control strain (RIB40). These analyses confirmed that translocated duplication of the targeted chromosomal region occurred in the regenerated strain; this strain was thereafter referred to as J4 and used in further analyses.

As described above, generating multiple translocated chromosomal duplications is theoretically possible. Therefore, we constructed a strain in which the targeted region of chromosome 2 was triplicated, and evaluated the effects of triplication on the phenotype of this strain. Initially, we removed the *pyrG* marker at the border of the translocated duplication region of chromosome 4 by homologous recombination between the chromosome and fragment used to remove *pyrG* (Additional file 1: Figure S3, top). Transformants were then selected on 1.2-M sorbitol-CZ plates containing 5-FOA, and the vector for introducing 3′*ΔpyrG* with the I-SceI recognition sequence was integrated near the end of chromosome 7 (Additional file 1: Figure S3, acceptor region). This was followed by selection using 5-FOA. The resulting triplication-containing parental strain (K1-IF5-2-5FOA) was cultured, and protoplasts were prepared for phenotypic analyses. Protoplasts were treated with I-SceI and PEG as described above. After incubation for 3 weeks at 30 °C, regenerated colonies were analyzed by PCR. After single-colony isolation (details are described in Additional file 1: Fig. S4), the strain containing homokaryotic nuclei with the translocated chromosomes was thereafter referred to as I-8 strain.

### Confirmation of translocated chromosomal duplications using array comparative genome hybridization (array CGH)

CGH was used to confirm the presence of translocated chromosomal duplication in the targeted chromosomal region. Briefly, genomic DNA isolated from RIB40 (control) and J4 strains (*Δku70* strain with translocated chromosomal duplication) was labeled with Cy dyes, hybridized on microarray slides at 65 °C for 24 h, and scanned with a laser-scanner. Comparison of the two strains revealed that the signal ratio of the probes in the 1.4-Mb region of chromosome 2 (AO090003001035–AO090003001558) was approximately two, and that of the probes located in the 308-kb region near the terminal end of chromosome 4 (AO090166000010–AO090166000123) was nearly zero (Fig. 3b). These observations confirmed the presence of translocated duplication of the targeted chromosomal region in the J4 strain (Fig. 3b and Additional file 1: Figure S1). The BN1-1 strain (*ku70 *+ strain with translocated chromosomal duplication) was also analyzed using CGH. Similar results were obtained (Additional file 1: Figure S2), indicating that translocated duplication of the targeted chromosomal region occurred successfully in the J4 and BN1-1 strains.

**Fig. 4:**
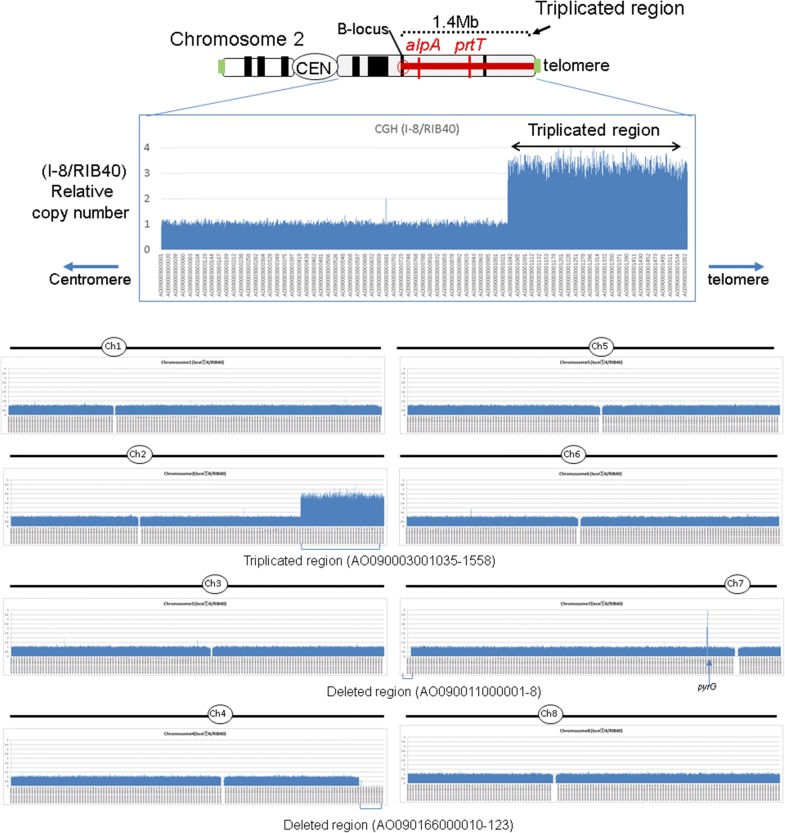
Confirmation of translocated duplication by array CGH. Results of array CGH indicate that the 1.4-Mb region of chromosome 2 was triplicated and translocated to the ends of chromosomes 4 and 7

Next, genomic DNA isolated from RIB40 (control) and I-8 strains (*Δku70* strain with translocated chromosomal triplication) was labeled with Cy dyes, hybridized on microarray slides, and scanned using a laser-scanner. As shown in Fig. 4, comparisons of triplicate (I-8) and control strains revealed a signal ratio of approximately 3 for the probes located in the 1.4-Mb region of chromosome 2 (AO090003001035–AO090003001558). Moreover, the signal ratios of the probes located in the 308-kb near-telomeric region of chromosome 4 (AO090166000010–AO090166000123) and in the 13-kb near-telomeric region of chromosome 7 (AO090011000001–AO090011000008) were nearly zero, indicating successful translocation of chromosome 2 to the end of chromosomes 4 and 7 in the I-8 strain (Fig. 4). These results indicate that multiple translocated duplications (such as translocated triplication) of the targeted chromosomal region were achieved in the I-8 strain.

### Gene expression analysis of translocated chromosomal duplication and triplication strains in solid-state culture

Segmental duplication of large chromosomal regions may upregulate numerous genes in the duplicated chromosomal region and cause phenotypic changes via gene dosage effect. To demonstrate the effects of chromosomal duplication on gene expression, we examined gene expression levels of the strains under conditions of solid-state fermentation. The J4 strain with a translocated duplication of a 1.4-Mb region of chromosome 2, I-8 strain with a translocated triplication of the 1.4-Mb region of chromosome 2, and RIB40 strain (control) were cultivated for 65 h at 30 °C in wheat bran medium; then, RNA was extracted and analyzed by gene expression microarrays. The ratios of upregulated genes in duplicated chromosomal regions were remarkably higher in strains with duplicated and triplicated chromosomes. As shown in Tables 1 and 2, gene expression was increased by 11% in the whole chromosomal region (1293 vs. 12010). Gene expression was increased by 37 and 64% in the strains with duplicated (166 vs. 446) and triplicated regions (284 vs. 446), respectively. These data indicate that segmental duplication and triplication of target chromosomal regions effectively increased transcription of resident genes. These data were summarized according to Clusters of Orthologous Groups (COGs) classification [19]. Per COGs classifications, the influence gene dosage effect on signal transduction mechanisms (T) was low with 25% increases in the J4 (2 vs. 8) and 38% increases in the I-8 (3 vs. 8) strains. The expression of genes categorized into the nucleotide transport and metabolism (F) group, per COGs classification, was significantly increased (5 vs. 5) in the duplicated region of the strains (Tables 1 and 2). In contrast, gene expression was decreased by 4 and 2.5% in the duplicated (17 vs. 446) and triplicated chromosomal regions, respectively. For the chromosomes in the J4 (duplicated) strain, sites of upregulated genes were identified using probes (Additional file 1: Figure S5), which clearly showed increased gene transcription in the duplicated chromosomal region. Approximately 10% of the genes located outside of the duplicated region (1127 vs. 11,564) were upregulated; several of these genes were markedly upregulated in the duplicated strain (Additional file 1: Figure S5), indicating the presence of regulatory factors in the duplicated region. The genes upregulated in the J4 strain are summarized in Tables 3, 4, 5 and 6. Twenty-three proteolytic genes, 8 amylolytic genes, and 23 xylanolytic genes, which were located outside of the duplicated region, were upregulated. Increased expression of proteolytic genes, located outside of the duplicated region, was observed in the J4 strain, suggesting that gene dosage effect exerted by *prtT* occurred in the duplicated region. Similarly, the upregulated expression of amylolytic genes indicated that gene dosage effect, exerted by *amyR*, occurred in the duplicated region. The expression of *xlnR* was not increased. However, we observed upregulated expression of genes related to xylanase, most of which were positively regulated by *xlnR* [20]. This indicate that *xlnR2* (AO090003001292) affected the expression of these genes. The expression of *prtT*, *amyR*, and *xlnR2* was upregulated in the duplicated region (Table 6). In contrast, 1252 genes located outside of the duplicated region were downregulated in the J4 strain, indicating that genes involved in negative regulation were also present in the duplicated region.

**Table 1:** Percent increase in the expression of upregulated genes in the duplicated chromosomal region of the J4 strain

COG description	Total	Up-regulated genes	%	Duplicated region	Up-regulated genes	%
[A] RNA processing and modification	176	3	2	8	1	13
[B] Chromatin structure and dynamics	38	2	5	4	2	50
[C] Energy production and conversion	371	56	15	9	3	33
[D] Cell cycle control, cell division, chromosome partitioning	101	10	10	5	2	40
[E] Amino acid transport and metabolism	453	65	14	18	9	50
[F] Nucleotide transport and metabolism	97	15	15	5	5	100
[G] Carbohydrate transport and metabolism	500	103	21	27	15	56
[H] Coenzyme transport and metabolism	133	13	10	4	3	75
[I] Lipid transport and metabolism	314	37	12	12	7	58
[J] Translation, ribosomal structure and biogenesis	277	5	2	6	3	50
[K] Transcription	180	4	2	9	3	33
[L] Replication, recombination and repair	172	11	6	7	2	29
[M] Cell wall/membrane/envelope biogenesis	98	11	11	5	1	20
[N] Cell motility	2	0	0	0	0	0
[O] Posttranslational modification, protein turnover, chaperones	390	19	5	12	6	50
[P] Inorganic ion transport and metabolism	195	21	11	9	2	22
[Q] Secondary metabolites biosynthesis, transport and catabolism	447	68	15	9	4	44
[R] General function prediction only	1370	186	14	51	18	35
[S] Function unknown	333	27	8	16	7	44
[T] Signal transduction mechanisms	310	26	8	8	2	25
[U] Intracellular trafficking, secretion, and vesicular transport	241	5	2	9	1	11
[V] Defense mechanisms	50	11	22	0	0	0
[W] Extracellular structures	3	0	0	0	0	0
[Y] Nuclear structure	6	0	0	0	0	0
[Z] Cytoskeleton	94	10	11	2	0	0
Unannotated	5659	585	10	211	70	33
Total	12,010	1293	11	446	166	37

*p < 0.02 and fold change > 1 indicate upregulated genes

% increase shows the percent ratio of upregulated genes to total genes in the whole genome or that of the genes in the duplicated region

**Table 2:** Percent increase in upregulated genes in the triplicated chromosomal region of the I-8 strain

COG description	Total	Up-regulated genes	%	Duplicated region	Up-regulated genes	%
[A] RNA processing and modification	176	9	5	8	7	88
[B] Chromatin structure and dynamics	38	3	8	4	3	75
[C] Energy production and conversion	371	42	11	9	5	56
[D] Cell cycle control, cell division, chromosome partitioning	101	13	13	5	5	100
[E] Amino acid transport and metabolism	453	60	13	18	14	78
[F] Nucleotide transport and metabolism	97	11	11	5	5	100
[G] Carbohydrate transport and metabolism	500	70	14	27	24	89
[H] Coenzyme transport and metabolism	133	13	10	4	3	75
[I] Lipid transport and metabolism	314	39	12	12	9	75
[J] Translation, ribosomal structure and biogenesis	277	8	3	6	6	100
[K] Transcription	180	10	6	9	8	89
[L] Replication, recombination and repair	172	14	8	7	5	71
[M] Cell wall/membrane/envelope biogenesis	98	10	10	5	3	60
[N] Cell motility	2	0	0	0	0	0
[O] Posttranslational modification, protein turnover, chaperones	390	21	5	12	10	83
[P] Inorganic ion transport and metabolism	195	18	9	9	4	44
[Q] Secondary metabolites biosynthesis, transport and catabolism	447	54	12	9	5	56
[R] General function prediction only	1370	138	10	51	24	47
[S] Function unknown	333	29	9	16	14	88
[T] Signal transduction mechanisms	310	21	7	8	3	38
[U] Intracellular trafficking, secretion, and vesicular transport	241	10	4	9	7	78
[V] Defense mechanisms	50	11	22	0	0	0
[W] Extracellular structures	3	0	0	0	0	0
[Y] Nuclear structure	6	0	0	0	0	0
[Z] Cytoskeleton	94	12	13	2	2	100
Unannotated	5659	537	9	211	118	56
Total	12,010	1153	10	446	284	64

*p < 0.02 and fold change > 1 indicate upregulated genes

% increase shows the percent ratio of upregulated genes to total genes in the whole genome or that of the genes in the duplicated region

**Table 3:** Upregulation of proteolytic genes located outside of the duplicated chromosomal region in the J4 strain

Systematic name	Relative gene^a^ expression	Description
AO090001000135	2.7	Mep20-metalloproteinase
AO090003000354	3.2	LapII, transferrin receptor and related proteins
AO090009000148	2.1	OpsA;aspartyl protease
AO090009000171	3.5	SPRT-like metalloprotease
AO090009000593	4.4	Metal-dependent amidase/aminoacylase/carboxypeptidase
AO090010000493	4.5	NpII-neutral protease II
AO090010000534	4.4	Serine carboxypeptidases (lysosomal cathepsin A)
AO090010000540	3.9	Aminoacylase ACY1 and related metalloexopeptidases
AO090011000036	9.7	Np I-neutral protease I
AO090011000052	4.7	LapI-leucine aminopeptidase
AO090011000235	3.0	TppA-tripeptidyl peptidase A
AO090012000022	2.9	Metal-dependent amidase/aminoacylase/carboxypeptidase
AO090012000080	2.4	Metal-dependent amidase/aminoacylase/carboxypeptidase
AO090012000706	4.0	Carboxypeptidase C (cathepsin A)
AO090020000288	3.1	Aminoacylase ACY1 and related metalloexopeptidases
AO090020000351	4.0	Serine carboxypeptidases (lysosomal cathepsin A)
AO090023000382	2.6	Carboxypeptidase C (cathepsin A)
AO090026000083	4.5	AorO-aorsin
AO090026000680	3.8	Serine carboxypeptidases (lysosomal cathepsin A)
AO090103000264	3.9	Predicted molecular chaperone distantly related to HSP70-fold metalloproteases
AO090138000101	4.0	Putative intracellular protease/amidase
AO090138000114	7.1	Meltrins, fertilins and related Zn-dependent metalloproteinases of the ADAMs family
AO090701000220	2.3	Carboxypeptidase C (cathepsin A)

^a^Relative gene expression in the J4 strain compared to that in the RIB40 strain was measured by gene expression array. *p* < 0.02

**Table 4:** Upregulation of amylolytic genes located outside of the duplicated region in the J4 strain

Systematic name	Relative gene^a^ expression	Description
AO090001000259	2.8	Beta-galactosidase
AO090001000492	2.6	Arabinogalactan endo-1,4-beta-galactosidase
AO090012000389	8.3	Beta-galactosidase/beta-glucuronidase
AO090023000944	2.5	Alpha-amylase
AO090038000471	2.5	Maltase glucoamylase and related hydrolases, glycosyl hydrolase family 31
AO090103000378	2.9	Alpha-amylase
AO090120000158	6.4	Beta-galactosidase
AO090701000558	2.2	Alpha-glucosidases, family 31 of glycosyl hydrolases

^a^Relative gene expression in the J4 strain compared to that in the RIB40 strain was measured by gene expression array. *p* < 0.02

**Table 5:** Upregulation of xylanolytic genes located outside of the duplicated region in the J4 strain

Systematic name	Relative gene^a^ expression	Description
AO090001000164	2.3	Protein with predicted nucleotide binding activity*
AO090001000383	6.9	Extracellular catechol oxidase*
AO090005000476	2.8	Beta-xylosidase
AO090005000531	3.6	Endoglucanase B; predicted secretion signal peptide*
AO090005000698	3.7	Beta-xylosidase*
AO090010000063	5.5	Sugar transporter (MFS family); transcriptionally induced by growth on xylose*
AO090011000141	3.6	Putative exoarabinase*
AO090023000001	6.7	AbfB-alpha-L-arabinofuranosidase B
AO090023000401	3.3	PglB-polygalacturonase B*
AO090038000426	4.1	Dehydrogenases related to short-chain alcohol dehydrogenases*
AO090038000631	3.5	xdhA1-Xylitol dehydrogenase*
AO090103000087	3.0	Putative endoglucanase precursor*
AO090103000268	6.2	Beta-xylosidase*
AO090103000326	2.5	Beta-1,4-xylanase
AO090103000423	2.8	XynF1, beta-1,4-xylanase*
AO090103000426	4.0	Shikimate 5-dehydrogenase*
AO090124000023	3.1	AbfA,;alpha-L-arabinofuranosidase
AO090701000885	3.1	As Abf-alpha-L-arabinofuranosidase*
AO090001000649	3.4	Alpha-glucosidases, family 31 of glycosyl hydrolases*
AO090011000140	3.6	Beta-glucosidase-related glycosidases*
AO090011000715	2.7	Ak eglA-Endoglucanase
AO090023000056	2.1	Endoglucanase B
AO090038000175	2.5	Endoglucanase

*Positively regulated by XlnR [20]

^a^Relative gene expression in the J4 strain compared to that in the RIB40 strain was measured by gene expression array. *p* < 0.02

**Table 6:** Upregulation of proteolytic, amylolytic, and xylanolytic genes, and expression of their respective regulatory genes, located in the duplicated region of the J4 strain

Systematic name	Relative gene^a^ expression	Description
AO090003001036	3.2	Alp, subtilisin-related protease
AO090003001208	2.0	AmyR
AO090003001209	2.5	AgdA, maltase glucoamylase and related hydrolases, glycosyl hydrolase family 31
AO090003001211	5.1	prtT
AO090003001258	2.4	Peptidase family M48
AO090003001292	3.0	XlnR2
AO090003001305	2.7	Alpha-D-galactosidase (melibiase)
AO090003001341	8.3	Endoglucanase
AO090003001497	2.1	Glycosidases
AO090003001511	2.3	Beta-glucosidase-related glycosidases

^a^Relative gene expression in the J4 strain compared to that in the RIB40 strain was measured by gene expression array. *p* < 0.02

### Enzymatic activity in translocated chromosomal duplication and triplication strains in solid-state culture

**Fig. 5:**
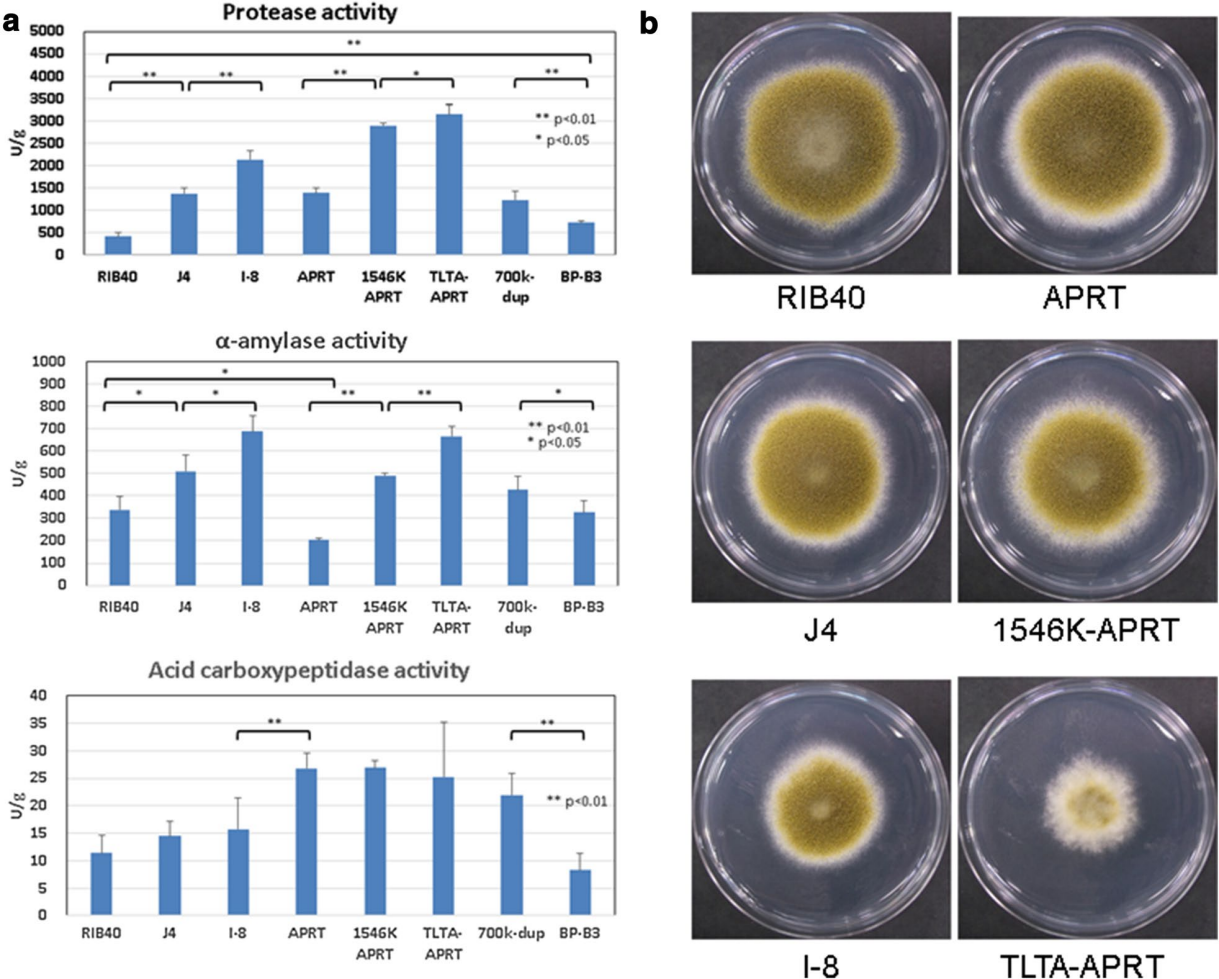
Phenotypes of translocated duplication strains. **a** Enzyme activity in strains bearing chromosomal duplication under solid-state culture conditions. Total protease, alpha-amylase, and acid carboxypeptidase activity in solid-state cultures; RIB40 (control), J4 (the strain bearing translocated duplication of chromosome 2), I-8 (the strain bearing translocated triplication of chromosome 2), APRT (*prtT* overexpression strain), 1546K-APRT (the strain bearing translocated duplication of chromosome 2 with *prtT* overexpression), TLTA-APRT (the strain bearing translocated triplication of chromosome 2 with *prtT* overexpression), Ao-700k-dup (the strain bearing tandem duplication of a 700-kb region of chromosome 2), BP-B3 (the strain bearing tandem duplication of a 9-kb region of chromosome 2). **b** Growth phenotypes of strains bearing chromosomal duplication cultured on CZ plates. The strains were inoculated onto 1.2 M sorbitol CZ plates and incubated at 30 °C for 7 days

To examine how chromosomal duplication and triplication affect phenotypes, we assessed enzymatic activity in translocated chromosomal duplication strains under solid-state conditions. We have previously shown increased activity of protease, amylase, and acid carboxypeptidase in strains with the 700-kb region (AO090003001003–AO090003001258) of the tandem duplication of chromosome 2 under conditions of solid-state cultivation on wheat bran media [12]. Therefore, we measured the activities of these enzymes in translocated duplication strains under solid-state culture conditions (Fig. 5a). Both translocated (J4) and tandem (700k-dup) chromosomal duplications led to increased activity of protease and amylase. Moreover, the activity of acid carboxypeptidase in the translocated duplication strain showed slightly higher levels than that in the wild-type strain (RIB40 control), but lower levels than that in the tandem duplication strain. A list of genes located in the overlapped region between J4 and 700k-dup is provided in Additional file 1: Table S2. We then examined enzymatic activity in the triplication strain I-8 under solid-state culture conditions. Protease activity was increased by more than fourfold in the triplication strain (I-8) compared to that in the control strain (RIB40), and was higher than those in the translocated and tandem duplication strains (J4 and 700k-dup) (Fig. 5a). The activity of amylase was also higher in the triplication strain (I-8) than in the duplication strains (J4 and 700k-dup), indicating that gene dosage showed increased phenotypic effects after multiple duplication of the chromosomal region. The BP-B3 strain, a tandem duplication strain bearing a 9-kb region (AO090003001033-AO090003001036) that included *alp* (gene encoding alkaline protease, AO090003001036) [21], showed 1.7-fold increase in protease activity compared to that of the control strain (Fig. 5a). The expression of *alp* and *prtT* in the duplication strains were examined by real-time PCR (Table 7).

**Table 7:** Quantitative expression of *alp* and *prtT* in strains bearing chromosomal duplication

Strain	*alp*			*prtT*		
	Relative^a^ quantity (dR)	Upper^b^ error bars	Lower^b^ error bars	Relative^a^ quantity (dR)	Upper^b^ error bars	Lower^b^ error bars
RIB40	1	0.58	0.37	1	0.26	0.21
J4	3.11	0.58	0.49	4.14	0.61	0.53
I-8	7.10	2.18	1.98	5.81	1.32	1.54
D2	2.84	1.16	0.71	4.28	1.75	1.08
BP-B3	1.84	1.47	0.70	2.19	1.67	0.92

^a^Relative quantity of gene expression was measured by real-time PCR

^b^Data are expressed as mean ± SD, and all experiments were conducted in triplicate

We then overexpressed the transcription factor *prtT* and examined its effects in duplicated and triplicated chromosomal translocation strains. PrtT regulates proteolytic enzymes in *A. oryzae* [22]. Our gene expression microarrays revealed moderate fivefold increases in *prtT* expression in duplication strains. These data suggest that *prtT* is rate-limiting, indicating increased protease activity in *prtT* over-expressing strains. The *prtT* overexpression vector pAPRT contains an *amyB* promoter and terminator, connected to the open reading frame of the *prtT* gene, and a *pyrG* marker. pAPRT in circular state was used to transform the RP1 strain (*pyrG* deletion strain), del 1546K4 strain (*pyrG* deletion strain derived from the J4 strain), and TLTAs11B strain (*pyrG* deletion strain derived from the I-8 strain). Single transformants showing clear large halos on casein plates, which indicates high protease activity [12], were selected for each *prtT* overexpression strain; overexpression of *prtT* was confirmed by real-time PCR (Table 8).

**Table 8:** Quantitative expression of *alp* and *prtT* in *prtT* overexpression strains

Strain	*alp*			*prtT*		
	Relative^a^ quantity (dR)	Upper^b^ error bars	Lower^b^ error bars	Relative^a^ quantity (dR)	Upper^b^ error bars	Lower^b^ error bars
RIB40	1	0.08	0.07	1	0.15	0.13
APRT	5.26	1.19	1.00	15.69	2.45	2.14
1546 K APRT	8.07	1.10	1.03	15.34	2.27	2.03
TLTA-APRT	17.31	1.52	1.35	18.33	2.34	2.05

^a^Relative quantity of gene expression was measured by real-time PCR

^b^Data are expressed as mean ± SD, and all experiments were conducted in triplicate

The expression of *prtT* was increased more than 10-fold in the APRT, 1546 K-APRT, and TLTA-APRT strains compared to that in the control strain (Table 8). Accordingly, the activity of protease in solid-state cultures (Fig. 5a) was approximately threefold higher in the APRT-transformed strain than in the RIB40 strain (wild type). This indicates that a single copy of *alp* was rate-limiting for protease activity under condition of *prtT* overexpression. Moreover, protease activity was similar in the 1546 K-APRT and TLTA-APRT strains and nearly sixfold higher than that in the RIB40 strain; this indicates that two copies of *alp* were sufficient for protease activity when *prtT* was overexpressed.

The APRT, 1546 K-APRT, and TLTA-APRT strains showed similarly increases in the activity of acid carboxypeptidase compared to that of the RIB40 strain. This suggests that acid carboxypeptidase genes are transactivated by *prtT,* but are not located in the duplicated chromosomal region. Single-copy integration of pAPRT in the APRT and TLTA-APRT strains, and two-copy integration of pAPRT in the 1546 K-APRT strain, were confirmed by quantitative PCR (Additional file 1: Figure S7).

The growth phenotypes of translocated duplication strains on CZ plates are presented in Fig. 5b. The strains were cultured on 1.2 M sorbitol-CZ plates at 30 °C for 7 days. The J4 strain showed a slight delay in growth compared to the growth of the RIB40 strain. The I-8 strain showed a slight delay in growth compared to that of the J4 strain. The growth rate of the strains gradually decreased as the copy number of the duplicated region from chromosome 2 increased. The deletion of a 308-kb region from chromosome 4, resulting from translocation in the J4 and I-8 strains, may have caused the delay in growth. The growth rates of the APRT and 1546 K-APRT strains were similar to those of the RIB40 and J4 strains, respectively. However, the TLTA-APRT strain showed a severe delay in growth compared to that of the I-8 strain. As shown in Table 8, the expression of *alp* was extremely high in the TLTA-APRT strain compared to that in the APRT or 1546 K-APRT strains. The expression of *alp* was originally high in the control strain (RIB40), suggesting that increased expression of *alp* caused growth delays in the TLTA-APRT strain.

## Discussion

In this study, we generated strains of *A. oryzae* in which a targeted chromosomal region was translocated and duplicated. CGH and PCR were used to confirm that targeted translocation indeed occurred. Because CGH is commonly used to detect chromosomal rearrangement in yeast. It was confirmed that the original genome of *A. oryzae* did not contain an I-SceI recognition sequence [17], indicating a low possibility of unexpected chromosomal rearrangement.

The strain with the translocated duplication of the targeted chromosomal region was obtained by treating protoplast cells with the I-SceI enzyme. No strains containing translocated duplication were generated in the absence of I-SceI, indicating that translocated chromosomal duplication in *A. oryzae* requires artificially introduced DSBs and depends on the break-induced replication mechanism. We previously obtained tandem chromosomal duplications from protoplasted cells without I-SceI-derived chromosomal DSBs in *A. oryzae* [12] and hypothesized that the tandem chromosomal duplications were generated by a nonallelic homologous recombination mechanism instead of classical unequal sister chromatid exchange in diploid budding yeast [23]. In addition, the time required for regenerating cells and frequency of regenerated colonies differed between tandem and translocated chromosomal duplications in *A. oryzae*. Specifically, tandem chromosomal duplications were produced after incubating protoplast cells for 5–7 days on regeneration plates, and the frequency of regenerated colonies from the protoplast cells was approximately 10^−7^ [12]. In contrast, strains with translocated chromosomal duplications were generated only after protoplast cell regeneration was conducted for more than 2 weeks, and the frequency of regenerated colonies from the protoplasted cell was approximately 10^−8^. To obtain translocated chromosomal triplications in regenerated state, nearly 3 weeks were necessary to regenerate the colonies, and most regenerated cells were heterokaryons containing both the original chromosome and translocated chromosome (Additional file 1: Figure S4). Hence, in the present study, single-colony isolation was necessary to obtain a pure translocated chromosomal triplication strain, indicating that more time is required for recombination to form translocated chromosomal regions in *A. oryzae*. These results indicate that tandem duplication of a targeted chromosomal region achieved in our previous study [12], and translocated duplication of a targeted chromosomal region achieved in the present study, depend on completely different recombination mechanisms and techniques.

In the present study, we generated duplication and triplication strains of *A. oryzae* chromosome 2 including *alp* and *prtT.* The strains bearing duplicated and triplicated chromosome 2 showed significant increases in the activity of protease under solid-state culture conditions.

However, protease activity in the BP-B3 strain (generated using tandem duplication of the 9-kb region, which included *alp* but not *prtT*) was less than those of the J4 and 700k-dup strains (generated using duplication of a chromosomal region containing both *alp* and *prtT*) (Fig. 5a). This indicates that duplication of both *alp* and *prtT*, or a structural gene and its regulatory gene, enhances the activity of protease. The duplicated region of chromosome 2 included the alpha-amylase gene and *amyR*, suggesting that amylase activity in the strains was enhanced via similar mechanism.

Oligonucleotide microarrays revealed increased gene expression with increasing amounts of genetic material in the strains containing duplicated and triplicated chromosome regions as compared to that of the control strain (Tables 1 and 2). Thus, the transcription levels of genes in the duplicated chromosomal regions were predominantly affected, and the resulting phenotypes were enhanced as the copy number increased. This suggests that segmental chromosomal duplication can be used to identify unknown gene functions based on phenotypic observations.

Protease activity was used to confirm the duplication stability of chromosome 2. Genome stability of *A. oryzae* strains in which translocated duplication was generated by mutagenesis was described in a United States patent US8900647B2. The genomes of the strains bearing translocated duplication were stable after 10 generation of subculture. Additionally, in our previous report [12], we showed that targeted tandem duplication of chromosome 2 remained stable after 5 rounds of subculture. Translocated duplication is more stable than tandem duplication because the possibility of losing the duplicated region via recombination is low.

Translocated duplication strains were generated from both *ku70 *+ and *ku70*- strains; the BN1-1 strain was derived from *ku70 *+ and J4 strain was derived from *ku70*− (Table 9). This suggests that non-homologous end-joining pathways involving Ku70/80 heterodimers are not important in the mechanism of translocated duplication. As shown previously, undesirable deletions or illegitimate recombinations were not observed in the combination of the *Δku70* strain and 5FOA counter selection [17]. This indicates that the *Δku70* strain of *A. oryzae* is suitable for *pyrG*-mediated targeted chromosomal duplication used previously [12] and in our present study. Therefore, it is possible to identify interesting phenotypes by constructing a chromosome duplication strain library that covers the whole genome and to detect unknown regulatory genes by screening and analyzing the library in *A. oryzae*.

**Table 9:** Strains used in the present study

*A. oryzae* RIB40 strain	Genotype	Source or reference
RIB40	Wild type	
Ku70RC7-2	*Δku70*, *ΔpyrG*	[10]
RP-1	*ΔpyrG*	[7, 8]
Ao-700k-dup	Tandem duplication of 700kb region of chromosome 2	[12]
B1036p5′ΔCTp3′Δ	*5′ΔpyrG*, *3′ΔpyrG* (parental strain of BN1-1)	Present study
ku70-B1036p5′ΔCTp3′Δ	*Δku70*, *5′ΔpyrG*, *3′ΔpyrG* (parental strain of J4)	Present study
BN1-1	Translocated duplication of 1.4 Mb region of chromosome 2 to chromosome 4	Present study
J4	*Δku70*, translocated duplication of 1.4 Mb region of chromosome 2 to chromosome 4	Present study
del1546K4	*Δku70*, translocated duplication of 1.4 Mb of region chromosome 2 to chromosome 4, *ΔpyrG*	Present study
K1-IF5-2-5FOA	*Δku70,* translocated duplication of 1.4 Mb region of chromosome 2 to chromosome 4, *5′ΔpyrG, 3′ΔpyrG* (parental strain of I-8)	Present study
I-8	*Δku70*, translocated duplication of 1.4 Mb region of chromosome 2 to chromosome 4 and chromosome 7	Present study
TLTAs11B	*Δku70*, translocated duplication of 1.4 Mb region of chromosome 2 to chromosome 4 and chromosome 7, *ΔpyrG*	Present study
APRT	pAPRT	Present study
1546 K-APRT	*Δku70*, translocated duplication of 1.4 Mb region of chromosome 2 to chromosome 4, pAPRT	Present study
TLTA-APRT	*Δku70*, translocated duplication of 1.4 Mb region of chromosome 2 to chromosome 4 and chromosome 7, pAPRT	Present study
BP-B3	Tandem duplication of 9 kb region of chromosome 2	Present study

## Conclusion

In this study, we achieved translocated chromosomal duplication and triplication of a 1.4-Mb targeted chromosomal region by directly introducing I-SceI meganuclease into *A. oryzae* protoplast cells. Strains with duplication and triplication of chromosome 2 showed substantial increases in the activity of protease and amylase. Gene dosage effects were enhanced by combining the structural gene and its regulatory gene, indicating that segmental duplications of chromosomes play important phenotypic roles in koji mold strains.

## Methods

### Strains, media, and transformation

Host strains included *A. oryzae* RIB40 (ATCC 42149), *A. oryzae* RP-1(*ΔpyrG*) [7, 8], and *A. oryzae* Ku70RC7-2 (*Δku70*, *ΔpyrG*) [10]. Positive selection of *pyrG*-deficient strains was performed on Czapek-Dox minimum medium (CZ) plates containing 20 mM uridine and 1.5 mg/mL 5-fluoroorotic acid (5-FOA). Solid-state cultivation was performed using medium containing 20% wheat bran and 80% water (wt/wt). Transformation of *Aspergillus* strains using protoplasts and PEG was conducted as previously described [17, 24]. Strains bearing translocated chromosomal duplication were obtained as follows. Approximately 1 × 10^8^ protoplasts were prepared from the host strain and maintained on ice for 30 min in a solution containing 0.05 mL of 1.2 M sorbitol, 0.02 mL of PEG, and 50 U of I-SceI endonuclease (New England Biolabs, Ipswich, MA, USA). After the addition of 0.07 mL PEG, the cells were incubated at room temperature for 1 h. Then, the cells were spread on regeneration plates containing 1.2 M sorbitol-CZ and incubated at 30 °C for 2–3 weeks. Regenerated colonies on regeneration plates were transferred to CZ plates for further analysis. Information on the genome of *Aspergillus oryzae* was collected from the genome database at the National Institute of Technology and Evaluation (http://www.bio.nite.g.o.jp/dogan/Top) and AspGD (http://www.aspergillusgenome.org). All oligonucleotide primers used in this study are listed in Additional file 1: Table S1.

### DNA techniques and expression analysis

Genomic DNA from *Aspergillus* strains was extracted as described previously [24]. RNA samples were prepared from mycelia that were inoculated on wheat bran medium and cultivated at 30 °C for 65 h. After cultivation, the mycelia were harvested and ground in liquid nitrogen using a mortar and pestle; RNA was extracted using Isogen reagent (Nippon Gene, Toyama, Japan). Further purification was performed using RNeasy Mini kits (Qiagen, Hilden, Germany) according to the manufacturer’s instructions. RNA was used in quantitative real-time PCR (RT-PCR) and gene expression microarrays. PCR amplification was performed using TaKaRa Ex Taq DNA polymerase (TaKaRa, Shiga, Japan) in a T100 thermal cycler (Bio-Rad, Hercules, CA, USA). Quantitative RT-PCR was performed in a MxPro3000P (Agilent Technologies, Santa Clara, CA, USA) using PrimeScript™ RT reagent with gDNA Eraser (Perfect Real Time) and SYBR^®^ *Premix Ex Taq*™ II (Tli RNase H Plus) (TaKaRa). The expression of *alp* and *prtT* were analyzed by comparative quantification using MxPro software version 4.10 (Agilent Technologies). AO090005000807 (ortholog of transcription factor TFIID) was used as normalizer, and primers are listed in Table S2. Oligonucleotide arrays were purchased from Agilent Technologies; the experimental protocol was detailed in a previous study [17, 25]. The CGH and gene expression array data obtained in the present study have been deposited into the NCBI Gene Expression Omnibus [26] and are accessible using GEO Series accession number GSE120604.

### Construction of vector used for partial deletion of *pyrG*

Construction of vectors used to create the 5′*ΔpyrG* and 3′*ΔpyrG* constructs was carried out in a manner similar to a method described previously [12]. The schematic of vector construction is shown in Additional file 1: Figure S6. The 5′*ΔpyrG* construct was *pyrG*-truncated by removing 631 bp from the 5′ end of *pyrG*, which included the promoter region. The 3′*ΔpyrG* construct was *pyrG*-truncated by removing 1236 bp from the 3′ end of *pyrG*, which included the terminator region. A 462-bp consensus region was present between 5′*ΔpyrG* and 3′*ΔpyrG*, and 3′*ΔpyrG* included an I-SceI site in the consensus region. To construct the 5′*ΔpyrG* vector, a 5′*ΔpyrG* vector backbone was constructed using the primers P499L, P509U, P1130U-IF5, and P1592L-IF5. Then, 5.8- and 0.5-kb fragments were amplified via PCR from the pPB9 plasmid (a pUC-based plasmid bearing a 3.0-kb fragment containing *pyrG*) [12] using the primer pairs P499L/P509U and P1130U-IF5/P1592L-IF5. The fragments were then ligated using an In-Fusion cloning kit (TaKaRa), and the basic construct for 5′*ΔpyrG* BP1130 was generated. To construct a vector for integrating 5′*ΔpyrG* at the B1036 site of chromosome 2, a 3-kb fragment was PCR-amplified from the genome of the RIB40 strain using primers B1036-U and B1036-L. Cloning of this fragment using a TA cloning kit (TOYOBO, Osaka, Japan) produced pB1036T, and pB1036T and BP1130 were PCR-amplified using the primers B1036-IFU and B1036IFL, and P363U and P3297L, respectively. The resulting two fragments were treated with DpnI and ligated using In-Fusion Cloning Kits (TaKaRa) to generate the pB1036pyr5′d vector. To construct the 3′*ΔpyrG* vector including the I-SceI site, a 3′*ΔpyrG* vector backbone was constructed using the primers P2788L, P2828U, P1130U-IF3, and P1592L-IF3. Then, a 5.8-kb fragment was amplified from the pPB9 plasmid [12] using the primer pair P2788L/P2828U, and a 0.5 kb-fragment was amplified from the pBP9-sceI (a pBP9-based vector bearing *pyrG*, including the I-SceI recognition site) [12] using the primer pair P1130U-IF3/P1592-IF3. The fragments were then ligated using an In-Fusion cloning kit (TaKaRa), and the basic construct for 3′*ΔpyrG* BP1130-I was generated. To construct the vector for integrating 3′*ΔpyrG* at the CT166 locus of chromosome 4, a 3-kb fragment of genomic DNA from the RIB40 strain was amplified using the primers ct166-U and ct166-L. This fragment was cloned using a TA cloning kit (TOYOBO) to prepare pCT166T. pCT166T was amplified via PCR using the primers ct166-IFU and ct166-IFL. The basic unit of 3′*ΔpyrG*, BP1130-I, was amplified using the primers P363U and P3297L. The resulting fragments were treated with DpnI and ligated into the vector using an In-Fusion Cloning Kits (TaKaRa) to obtain pCT166pyr3ʹd. The fragment for removing *pyrG* from the J4 strain was constructed as follows: a 2-kb amplification fragment and a 2.3-kb fragment were amplified from RIB40 genomic DNA using the primer pairs TL-B5626L and TL-B3616U-IF, and TL-ct3685L-IF and TL-ct1388U, respectively. Fragments were ligated using In-fusion cloning kits (TaKaRa) to obtain the vector for removing *pyrG* from the J4 strain. To construct the vector for integrating 3′*ΔpyrG* at the S11 locus of chromosome 7, a 1.3-kb fragment of genomic DNA from the RIB40 strain was amplified using the primers S11-U and S11-L. This fragment was cloned using a TA cloning kit (TOYOBO) to prepare pS11T. pS11T was amplified via PCR using the primers S11-IFU and S11-IFL. The basic unit of 3′*ΔpyrG*, BP1130-I, was amplified using the primers P363U and P3297L. The resulting fragments were treated with DpnI and ligated into the vector using In-Fusion Cloning Kits (TaKaRa) to obtain pS11pyr3ʹd. The fragment for removing *pyrG* from the I-8 strain was constructed as follows: a 2.8-kb fragment was amplified using the primers TL-B3616U-IF-S11 and B6217L, and a 2.7-kb fragment was amplified using the primers TL-S11-14389U-IF-B and S11-17060L. The two PCR products were then ligated using In-Fusion Cloning Kits (TaKaRa) to obtain the vector for removing *pyrG* from the I-8 strain. Details of *pyrG* removal using homologous fragments were described previously [10].

### Construction of parental strain for strains bearing translocated chromosomal duplication

To construct parental strains for generating strains with translocated chromosomal duplication, RP-1 (*ΔpyrG*) and ku70RC7-2 (*Δku70*, *ΔpyrG*) were transformed with pB1036pyr5′d. After confirming vector integration at target sites, transformants were subjected to selection using 5-FOA, and strains with 5′*ΔpyrG* at the target site of chromosome 2 were isolated (Additional file 1: Figure S6B). The strains B1036pyrG5′*Δ* and ku70-B1036pyrG5′*Δ* were transformed with pCT166pyr3′d. Strains with pCT166pyr3′d integrated at the target site were selected using 5-FOA to obtain parental strains for generating the strains B1036p5′*Δ*CTp3′*Δ* and ku70-B1036p5′*Δ*CTp3′*Δ* containing translocated chromosomal duplication (Additional file 1: Figure S6C). Construction of parental strain for translocated triplication was conducted as follows. The del1546 K strain, obtained by removing *pyrG* from chromosome 4 of the J4 strain (Additional file 1: Figure S4), was transformed with pS11pyr3ʹd. After confirming vector integration of chromosome 7, transformants were subjected to selection using 5-FOA. The strains with 3′*ΔpyrG* at the target site of chromosome 7 (K1-IF5-2-5FOA) were isolated (Additional file 1: Figure S4).

### Construction of *prtT* overexpression strains

The *prtT* overexpression vector pAPRT was constructed as follows. A 3.7 kb *amyB* fragment, amplified from RIB40 genome DNA using the primers amyU and amyl, was cloned by a TA cloning kit (TOYOBO) to generate pAmyTA. A 6.7-kb fragment, amplified from pAmyTA using the primer pair Amy118UIF/TA816UIF, and a 2.9-kb *pyrG* fragment amplified from pBP9 [12] using the primer pair P363U/P3229L, were fused using an In-Fusion-Cloning Kit (TaKaRa) to obtain pAmyPYR. A 7.6-kb fragment amplified from pAmyPYR, and a 2.1 kb fragment containing *prtT* ORF and amplified from RIB40 genomic DNA using the primer pair prtTstU/prtTteL, were fused using an In-Fusion-Cloning Kit (TaKaRa) to obtain the *prtT* overexpression vector pAPRT. The RP1, del1546K4, and TLTAS11B strains were transformed with pAPRT in a circular state to obtain the APRT, 1546 K-APRT, and TLTA-APRT strains, respectively. The copy numbers of the pAPRT vector integrated in the strains were estimated by quantitative PCR (Additional file 1: Figure S7). The APRT and TLTA-APRT strains contained one copy of APRT. The 1546 K-APRT strain contained two copies of pAPRT. Determining the copy number of pAPRT in the strains via Southern blot was difficult. This is because *A. oryzae* strains originally contain three copies of amylase genes, and the duplicated chromosomal region contained *prtT* and amylase genes. The results of PCR suggested that one copy of pAPRT in the 1546 K-APRT strain was integrated at one of the amylase loci (data not shown). The other copy of pAPRT appeared to be randomly integrated in the strains.

### Solid-state cultivation and measurements of enzyme activity

Solid-state cultures were generated by inoculating 1 × 10^8^ conidiospores into 5 g of wheat bran medium in a 150-mL Erlenmeyer flask and incubating at 30 °C for 4 days. Water (50 mL) was then added to the flask and extracted after shaking. The liquid fraction was filtered through filter paper and used as sample extract. Protease activity was determined by mixing the substrate and 2% milk casein (pH 7) with sample extracts and incubating at 30 °C for 20 min. Reactions were stopped using trichloroacetic acid, and quantities of liberated amino acids were measured at 660 nm using a tyrosine standard. One unit (U) was defined as the amount of enzyme yielding 1 µg of tyrosine per min at 30 °C and at pH 7. The activity of alpha-amylase was determined using Alpha-Amylase Activity kits (Kikkoman Biochemifa, Minato-ku, Japan) according to the manufacturer’s instructions. Briefly, reaction buffer containing 100 mM acetate (pH 5), 2 mM 2-chloro-4 nitrophenyl 6^5^-azido-6^5^-deoxy-beta-maltopentaocid, 50 U/mL glucoamylase, 6 U/mL beta-glucosidase, 50 mM NaCl, and 2 mM CaCl_2_ was mixed with 300 volumes of diluted sample extract and incubated for 5 min at 37 °C. The reactions were stopped using trichloroacetic acid, and liberated 2-chloro-4 nitrophenol contents were determined at 400 nm. One unit (U) was defined as the amount of enzyme yielding 1 µmol of 2-chloro-4 nitrophenol per min.

The activity of acid carboxypeptidase was determined using Acid Carboxypeptidase Assay kits (Kikkoman Biochemifa) according to the manufacturer’s instructions. The reaction buffer containing 50 mM acetate (pH 3), 0.5 mM Cbz-Tyr-Ala (carboxybenzoxy-L-tyrosyl-l-alanine), and 5 mM NAD was mixed with 30 volumes of diluted sample extract and incubated for 10 min at 37 °C. The reaction was stopped using 0.5 M Tris–HCl buffer (pH 8.5) containing 5 mM WST-8 and 13 U of alanine dehydrogenase, and the mixture was incubated at 37 °C for 20 min. Subsequently, 0.5 mM 1-methoxy-5-methylphenazinium methylsulfate was added to reaction mixture and incubated at 37 °C for 10 min. Quantities of liberated NADH were measured at 460 nm. One unit was defined as the amount of enzyme that liberated 1 µmol of l-alanine from Cbz-Tyr-Ala per min.

## Additional file

**Additional file 1.** Supplementary figures and tables.
