# Supplementary material for: Translocated duplication of a targeted chromosomal segment enhances gene expression at the duplicated site and results in phenotypic changes in Aspergillus oryzae
Source: Fungal Biol Biotechnol. 2018 Oct 3;5:17. doi: 10.1186/s40694-018-0061-6 (PMC6171174; doi:10.1186/s40694-018-0061-6)

**Supplementary materials**

**Translocated duplication of a targeted chromosomal segment enhances gene expression at the duplicated site and results in phenotypic changes in *Aspergillus oryzae***

Tadashi Takahashi*, Masahiro Ogawa, Atsushi Sato, Yasuji Koyama

*Noda Institute for Scientific Research, 399 Noda, Noda City, Chiba Pref 278-0037, Japan*

*Corresponding author

E-mail: ttakahashi@mail.kikkoman.co.jp

Supplementary figure S1


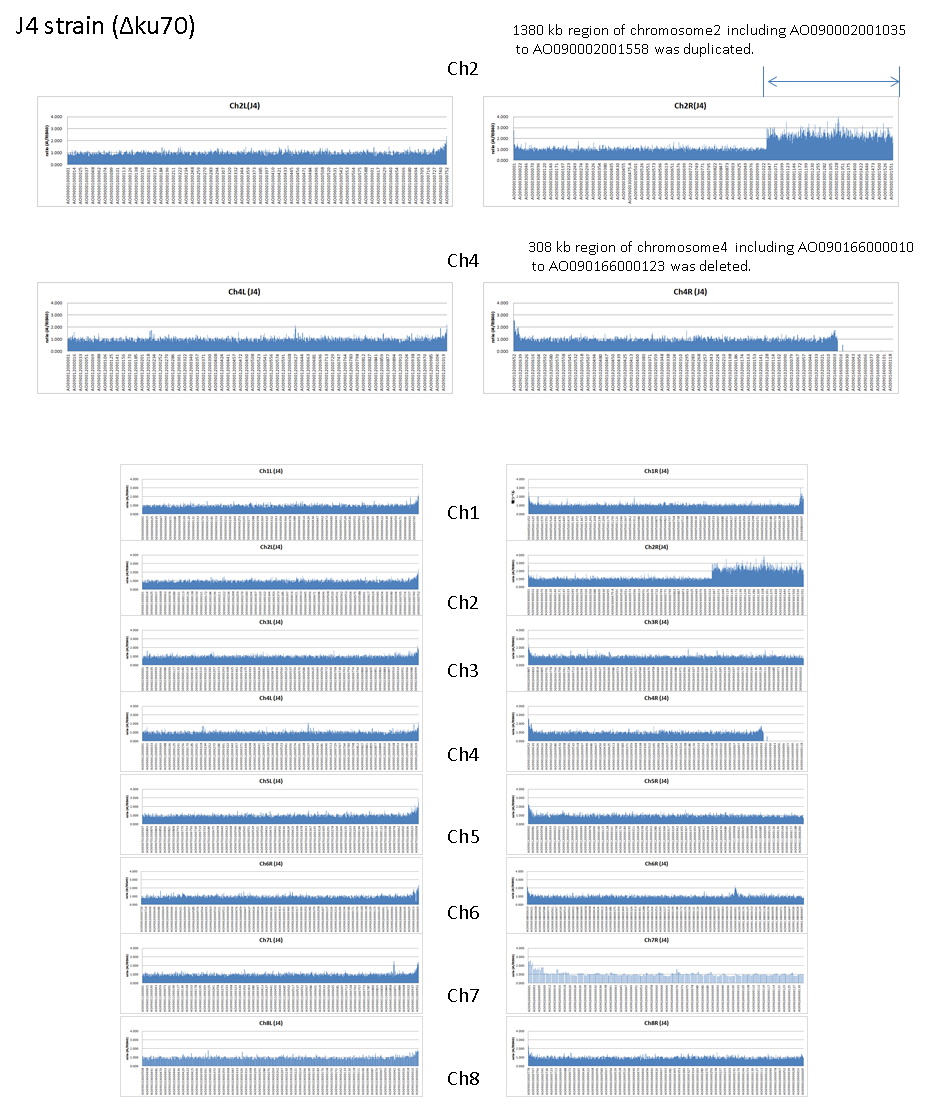


**Figure S1. Confirmation of translocated duplications in the J4 strain by comparative genome hybridization (CGH) arrays**

Supplementary figure S2


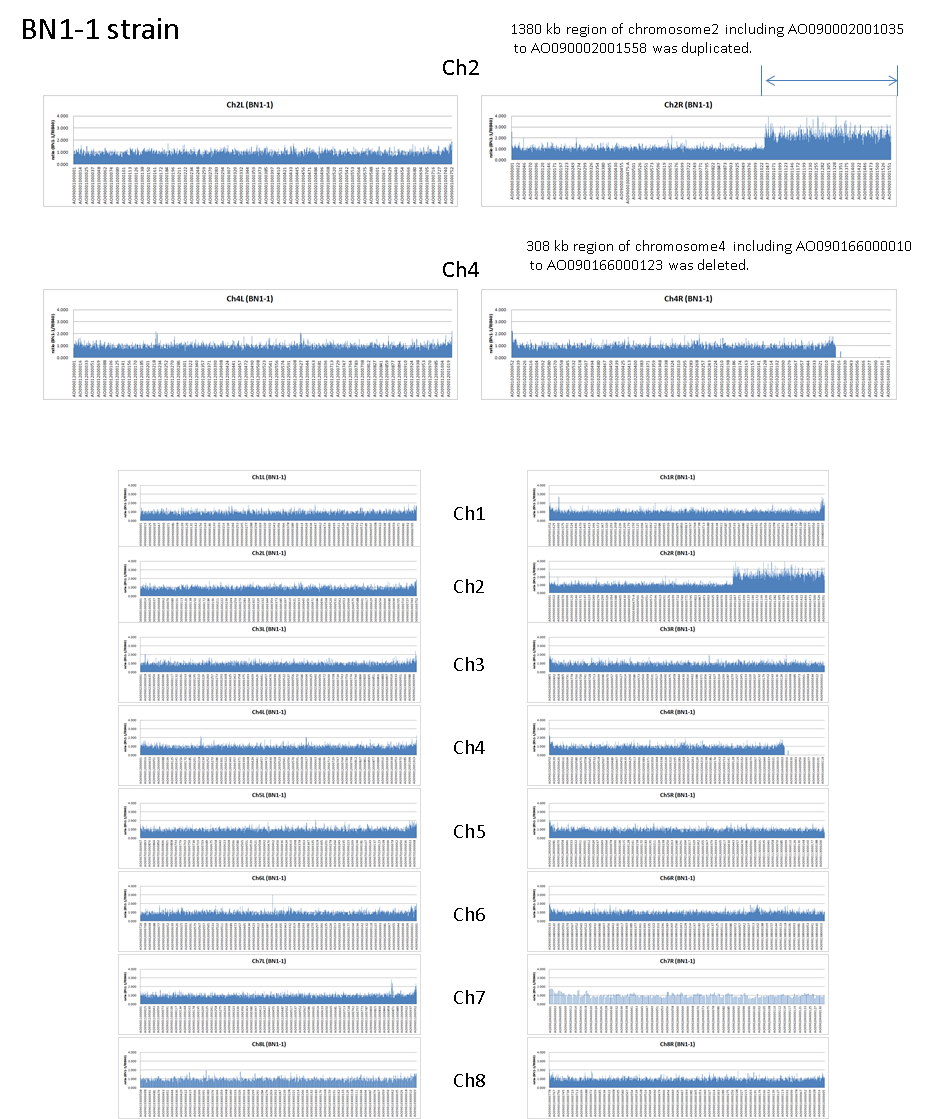


**Figure S2. Confirmation of translocated duplications in the BN1-1 strain by comparative genome hybridization (CGH) arrays**

Supplementary figure S3


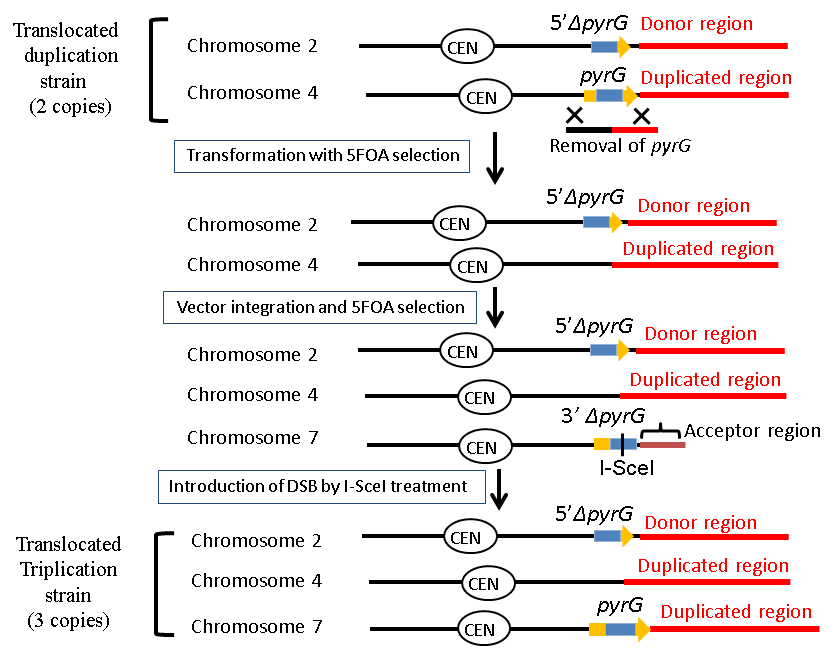


**Figure S3. Schematic of methods used for translocated triplication of chromosome 2 in *A. oryzae*.** *pyrG* from translocated duplication to chromosome 4 was removed by homologous recombination and selection using 5-fluoroorotic acid (5-FOA). The vector for 3′*Δ pyrG* with the I-SceI recognition sequence was integrated into chromosome 7 of the resulting *pyrG* strain. After 5-FOA selection, the parental strain for chromosome 2 triplication was constructed. Double-strand breaks (DSB) were introduced at the 3′*ΔpyrG* gene in chromosome 7 using I-SceI treatment, and the translocated triplication strain was obtained.

Supplementary figure S4


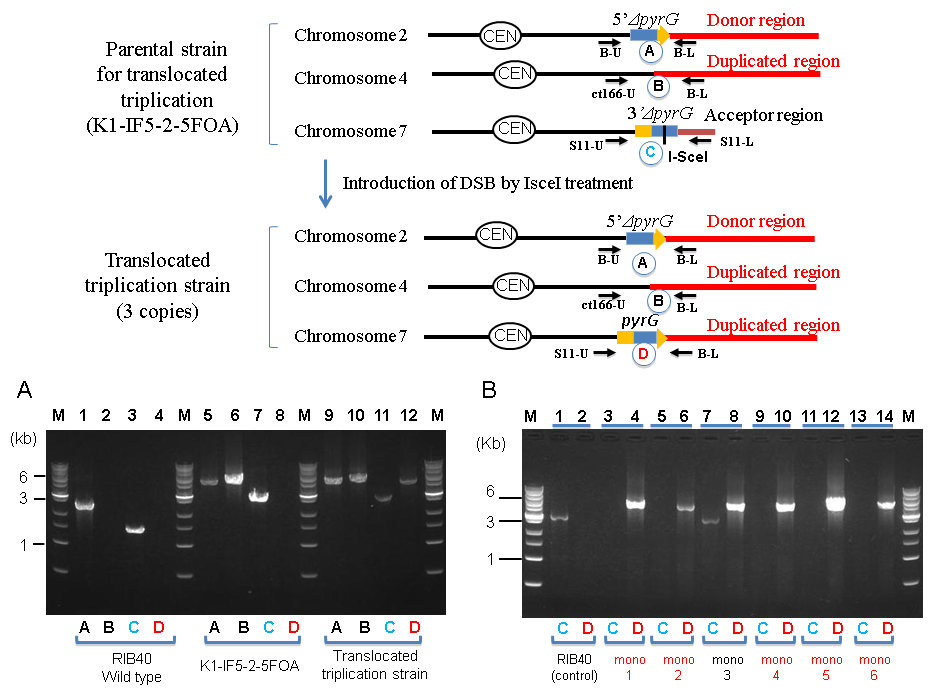


**Figure S4. Construction and confirmation of translocated triplication strain by PCR**

Translocated triplication of the strain was confirmed using primers targeting both ends of the duplication border region.

**A. Confirmation of translocated triplication.** Lane 1. A, B-U, B-L; Lane 2. B, ct166-U, B-L; Lane 3. C, S11-U, S11-L; Lane 4. D, S11-U, B-L; (Lanes 1–4, RIB40 wild-type); Lane 5. A: B-U, B-L, Lane 6. B:ct166-U, B-L Lane 7. C:S11-U, S11-L, Lane 8. D:S11-U, B-L (Lanes 5–8: K1-IF5-2-5FOA), Lane 9. A: B-U, B-L, Lane 10. B:ct166-U, B-L Lane 11. C:S11-U, S11-L, Lane 12. D:S11-U, B-L (Lanes 9–12: strain bearing translocated triplication). **B.** **Purification of homokaryotic strains of translocated triplication.** Lanes 1, 3, 4, 5, 7, 9, 11, and 13, C, S11-U, S11-L; Lanes 2, 4, 6, 8, 10, 12, and 14, D, S11-U, B-L (Lanes 1–2, RIB40; Lanes 3–4, mono1; Lanes 5–6, mono2; Lanes 7–8, mono3; Lanes 9–10, mono4; Lanes 11–12, mono5; Lanes 13–14, mono6).

(Supplementary Figure S4). Amplification of a DNA fragment using the primers D (Figure S4A, lane 12) and C (Figure S4A, lane 11) indicated that the regenerated colony was heterokaryotic, containing nuclei with the translocated chromosome and other nuclei with the original chromosome. To isolate homokaryotic strains, spores from the colony were spread on a casein plate, and six single colonies with large and clear halos were isolated. Subsequent PCR analyses revealed that five of the six colonies were homokaryotic strains with only translocated chromosomes (Figure S4B; mono1, mono2, mono4, mono5, and mono6), and the isolated mono1 colony was subjected to array CGH analyses as conducted for the I-8 strain.

Supplementary figureS5


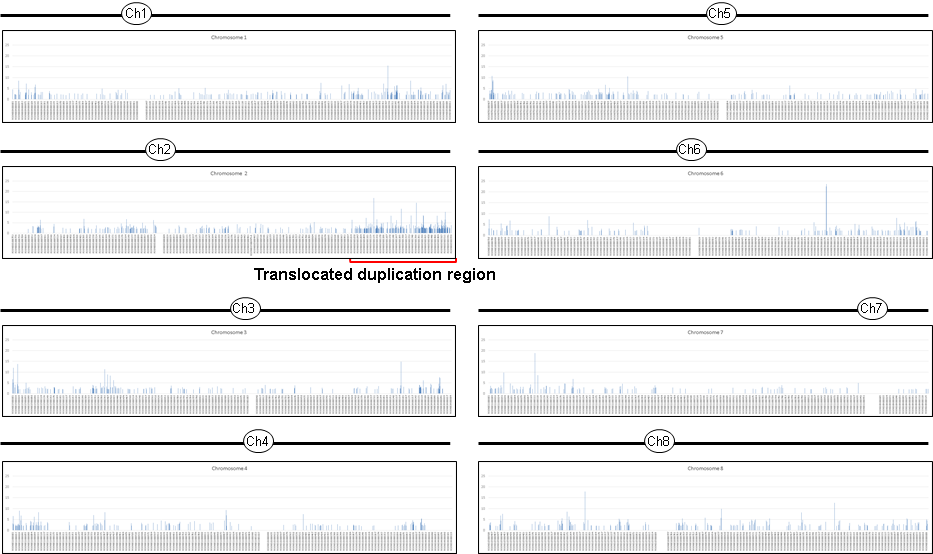


**Figure S5. Gene expression changes in a strain bearing translocated duplication.** Genes upregulated by more than 2-fold compared to the control strain, evaluated by expression microarrays, are presented. The transcription of the duplicated site in the J4 strain was increased by more than 2-fold compared to that in the control strain.

Supplementary figureS6


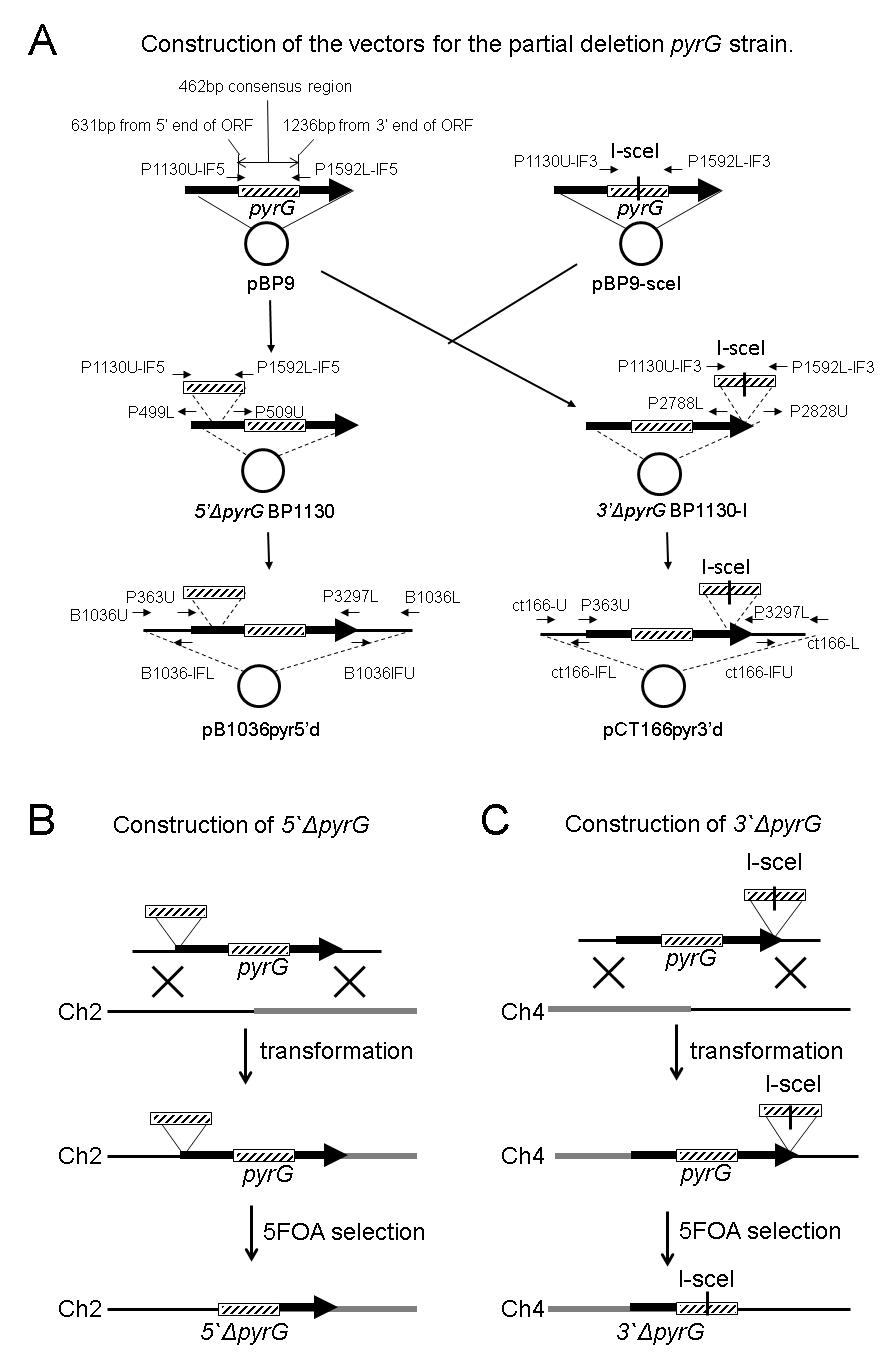


FigureS6. Schematic representation of vector construction for partial deletion in the *pyrG* strain.

Supplementary figureS7


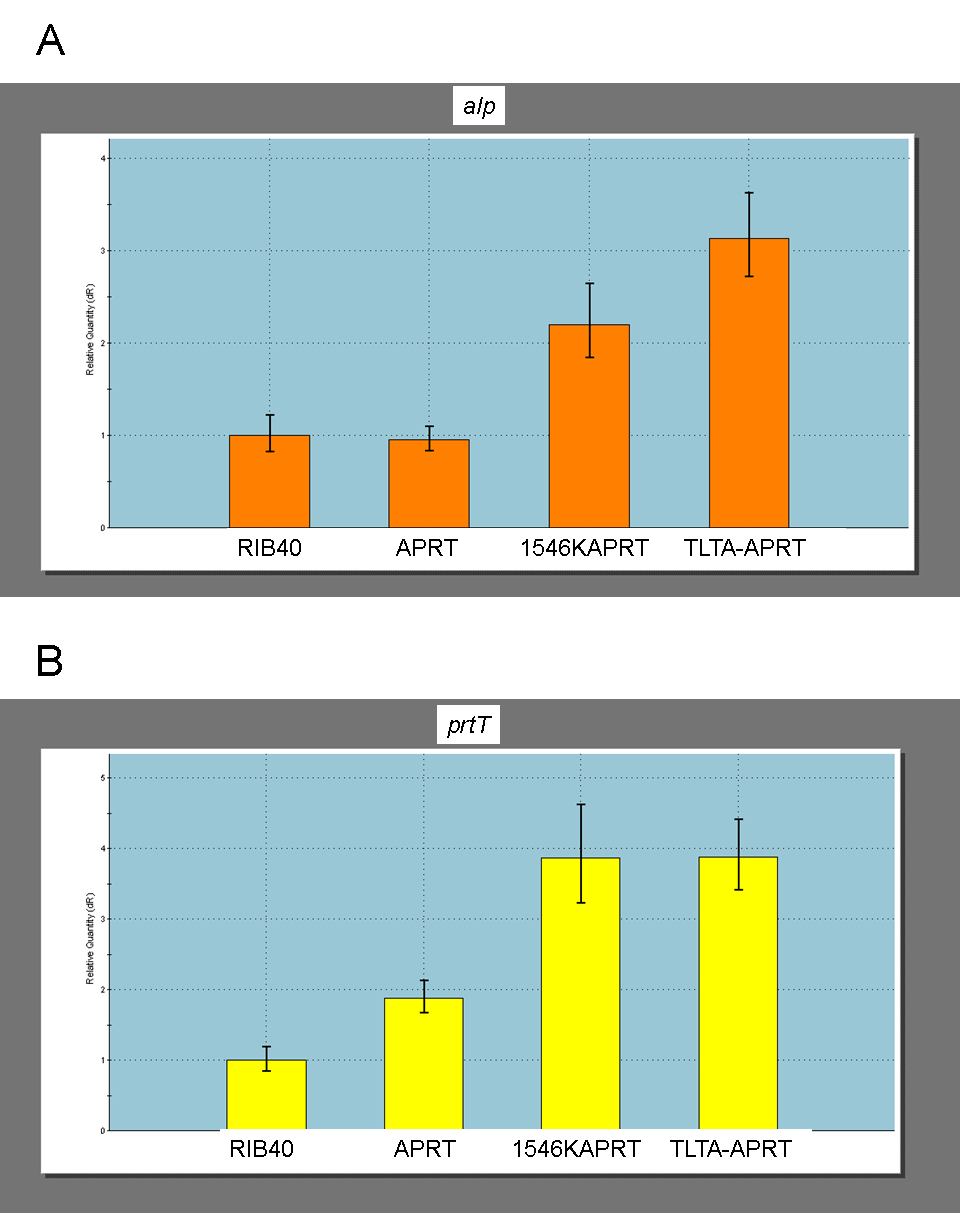


**FigureS7. Confirmation of copy number of *alp* and *prtT* in the strains. A.** Relative copy numbers of *alp* and *prtT* in the strains were determined by quantitative PCR.

Table S1. Oligonucleotide primers used in this study.


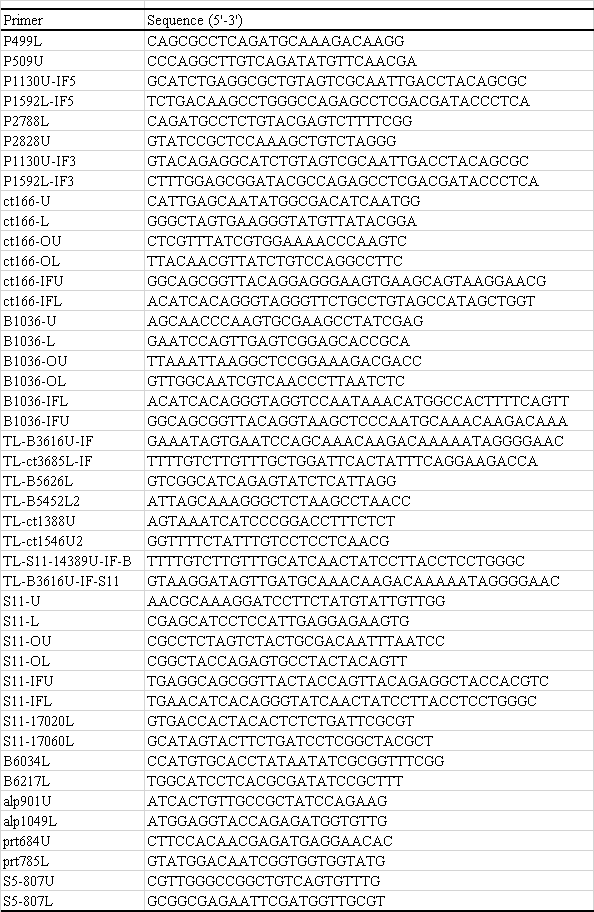


Table S2. A list of genes in the duplicated region overlapped between J4 (the strain bearing translocated duplication) and D2 (the strain bearing tandem duplication)


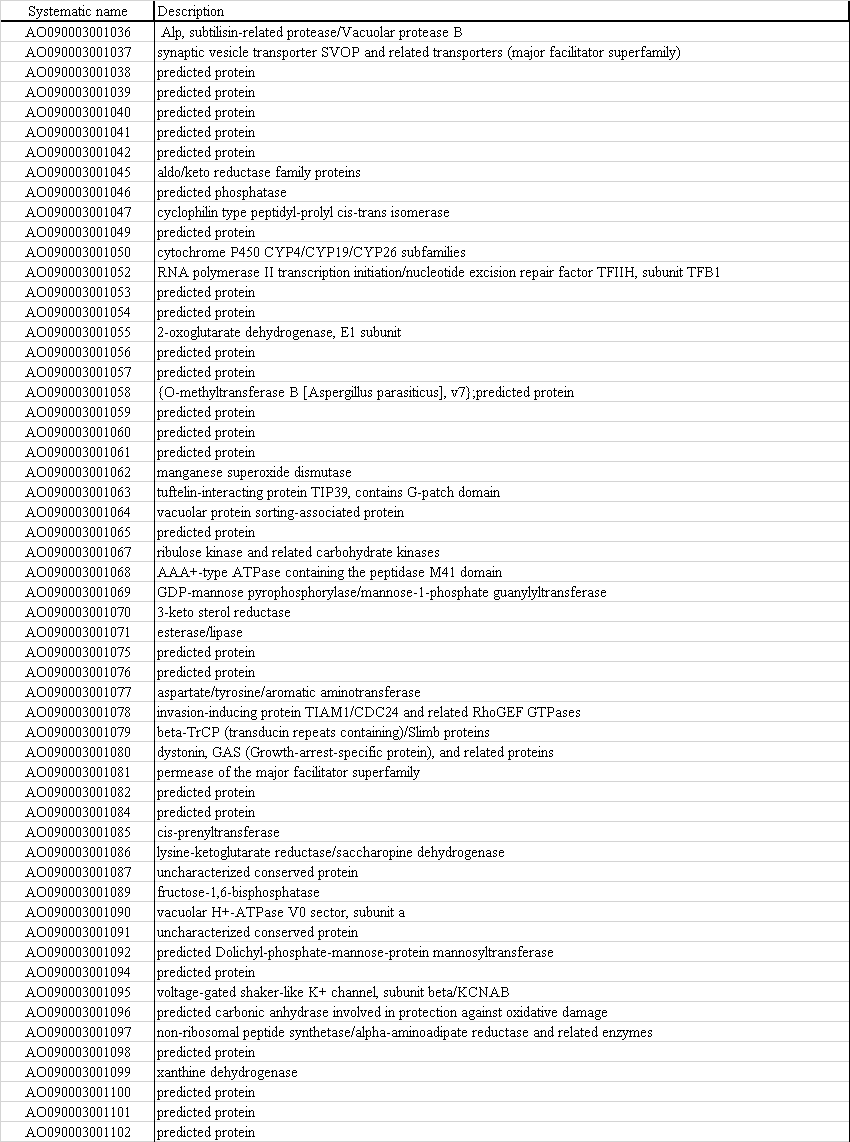


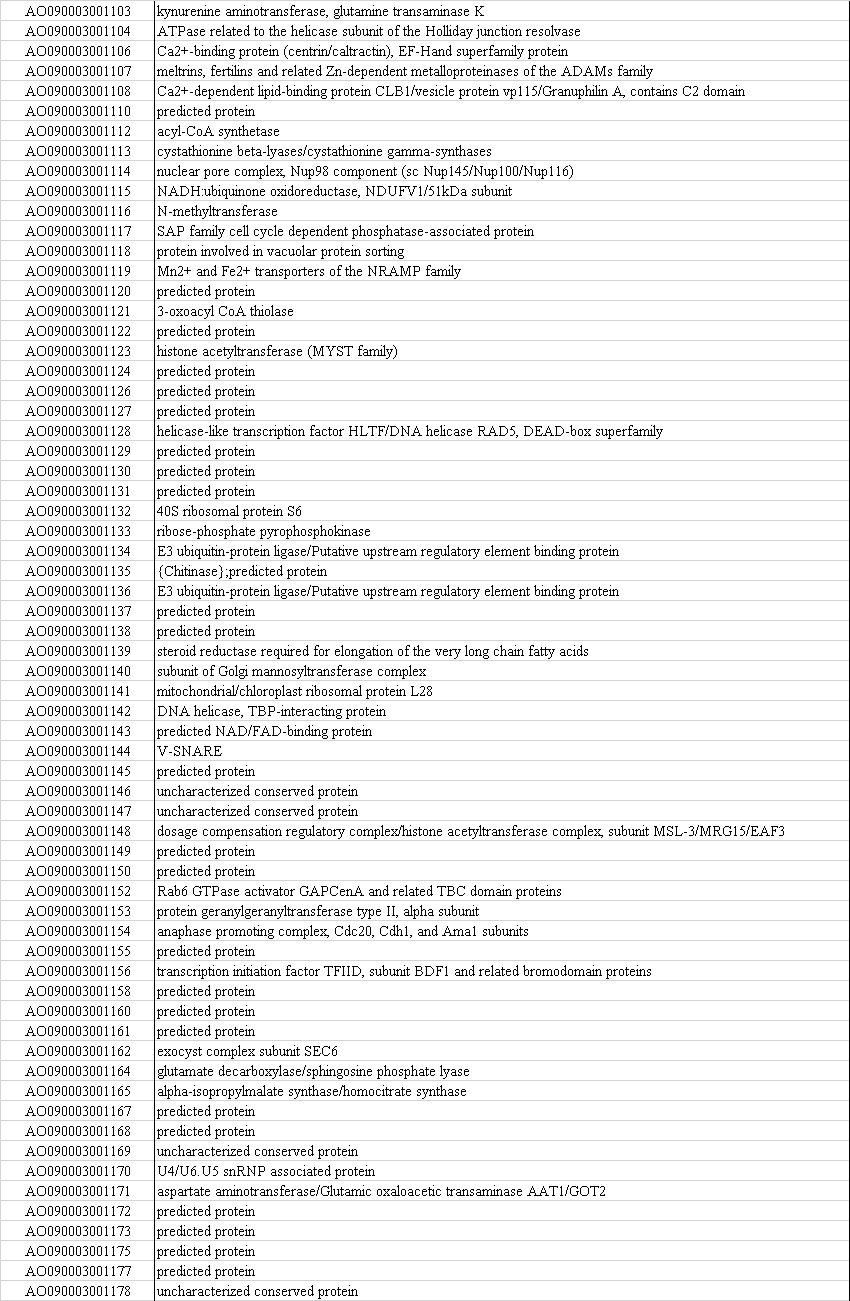

Supplement: Supplementary file 1 — Additional file 1. Supplementary figures and tables. [file 40694_2018_61_MOESM1_ESM.doc]
